# Determinants of Radiation Exposure in Middle Meningeal Artery Embolization for Chronic Subdural Hematoma: A Single-Center Cohort Study

**DOI:** 10.1007/s00062-026-01662-4

**Published:** 2026-04-27

**Authors:** Gregor Peter, Lukas Meyer, Luca Meucci, Felix Schlicht, Helge Kniep, Alexander Heitkamp, Christian Heitkamp, Laurens Winkelmeier, Bogdana Tokareva, Vincent Geest, Maxim Bester, Jens Fiehler, Matthias Bechstein, Christian Thaler

**Affiliations:** Department of Diagnostic and Interventional Neuroradiology, University Medical Center Hamburg-Eppendorf, Hamburg, Germany

**Keywords:** Middle meningeal artery embolization, Chronic subdural hematoma, Radiation dose, Transradial access, Embolic agents

## Abstract

**Purpose:**

To identify patient- and procedure-related factors associated with radiation exposure during middle meningeal artery (MMA) embolization for chronic subdural hematoma (cSDH), with particular focus on embolic agent (particle vs. liquid) and arterial access route (transradial vs. transfemoral).

**Methods:**

In this retrospective single-center cohort study, we included all patients who underwent MMA embolization for cSDH between February 2021 and October 2025. MMA embolization was performed by fourteen experienced board-certified neuroradiologists. The primary outcome was dose area product (DAP, Gy · cm^2^). Univariable and multivariable linear regression analyses were performed to assess associations between DAP and age, sex, access route, treated side (unilateral vs bilateral), embolic agent, anesthesia, interventionalist, and guiding catheter.

**Results:**

A total of 112 patients (median age 81 years; 20% female) were included; 69 (62%) underwent embolization with particles and 43 (38%) with liquid agents. Transradial access was used in 17 (15%) and transfemoral access in 95 (85%) procedures. Overall median (IQR) DAP was 74.7 Gy · cm^2^ (55.6–102.4), with 65.5 Gy · cm^2^ (50.6–82.0) for unilateral and 107.6 Gy · cm^2^ (84.1–161.6) for bilateral embolization. Univariable analyses showed no significant association between DAP and age, sex, embolic agent, access route, anesthesia, guiding catheter, or interventionalist. In multivariable analysis, unilateral right and left MMA embolization remained independently associated with significantly lower DAP compared with bilateral procedures, while embolic agent and access route were not significantly associated with radiation exposure.

**Conclusion:**

In MMA embolization for cSDH, radiation exposure is primarily driven by procedural extent, with higher DAP in bilateral than in unilateral embolization, whereas the choice of embolic material and arterial access route is not associated with radiation dose. These findings support selecting embolic agents and access routes based on anatomical and clinical considerations rather than radiation concerns.

## Introduction

Recent studies reported the benefit of middle meningeal artery (MMA) embolization to reduce the recurrence of chronic subdural hematoma (cSDH), supporting its growing role alongside surgery [1–3]. As clinical adoption expands, the cumulative radiation dose associated with these procedures will inevitably increase and affect both patients and operators [4]. To ensure safe application of particles or liquid embolics, continuous fluoroscopy is necessary to exclude inadvertent embolization via dangerous collaterals or retrograde reflux. This results in an increased fluoroscopy time and overall radiation dose. Although modern neurointerventional systems incorporate advanced dose-reduction technologies, systematic dose optimization remains a core element of procedural safety and quality assurance [5–7].

Despite increasing use [8], key technical aspects of MMA embolization remain under discussion, notably the choice of embolic agent (liquid vs particulate) and the arterial access route (transradial vs transfemoral) [9]. These decisions can influence catheter stability, positioning of the microcatheter, imaging requirements, and workflow efficiency, thereby affecting fluoroscopy time and dose area product [10]. Existing evidence is limited and heterogeneous with respect to how material selection and access route translate into radiation exposure, and direct comparisons under standardized conditions are scarce [11].

Accordingly, the aim of this study was to identify patient- and procedure-related factors associated with radiation exposure, in particular the comparison between liquid and particle embolic agents as well as transradial and transfemoral access route. We hypothesized that neither the arterial access route nor the embolic agent used is associated with higher radiation exposure after adjustment for patient- and procedure-related factors.

## Material and Methods

The study was approved by the ethics committee of [BLINDED FOR PEER REVIEW] in accordance with the Declaration of Helsinki. Patient informed consent was waived due to the retrospective study design. This study followed the Strengthening the Reporting of Observational Studies in Epidemiology (STROBE) reporting guideline for cohort studies.

### Study Population

This retrospective single-center study included all patients who received MMA embolization for chronic subdural hematoma at the [BLINDED FOR PEER REVIEW] between February 2021 and October 2025. Inclusion criteria were: age > 18 years, history of chronic subdural hematoma, consent by the patient or an authorized representative for the procedure.

#### Procedural Details

Decision for MMA embolization was made by an interdisciplinary team of neurosurgeons and neuroradiologists. All patients received surgery treatment, which was performed before MMA embolization. The MMA embolization was performed by fourteen experienced board certified neuroradiologists during the routine working hours (8 am to 6 pm). The choice between transfemoral (TFA) and transradial (TRA) access was made individually by each treating interventionalist. MMA embolization was performed using either liquid embolics (ethylene vinyl alcohol copolymer) or particles. The type of anesthesia was determined on a case-by-case basis, whereby general anesthesia was preferred for liquid embolics and local anesthesia for particles. If general anesthesia was not performed when using liquid embolics, inta-arterial lidocaine and intravenous piritramide were administered. Following arterial puncture and placement of a 6 or 7 French sheath, the ipsilateral internal carotid artery (ICA) was initially catheterized, and an angiographic series was performed to visualize the intracranial arteries, particularly to assess the origin of the ophthalmic artery. Subsequently, the guiding catheter was placed in the external carotid artery, and the origin of the MMA was visualized. The MMA was then selectively catheterized using a microcatheter and microwire, and a selective angiogram of the MMA was performed through the microcatheter. After excluding collaterals to the ophthalmic artery or cerebral supplying arteries, the catheter was positioned as distally as possible in the frontal or parietal branch when using liquid embolic agents, whereas with the use of particles, the catheter was placed more proximally, but still distal to the petrosquamous branch. The application of liquid embolic agents or particles was performed under continuous fluoroscopy. Finally, after successful embolization, an angiogram from the common carotid artery was performed. All MMA embolizations were performed using a biplane flat panel angiographic system (Azurion 7 B20/15, Philips, Amsterdam, Netherlands).

### Primary Outcome Parameters

Primary outcome parameter was the dose area product (DAP, mGy • cm^2^, converted to Gy • cm^2^). DAP was taken from the dose card which was generated automatically for each examination and sent to our Picture Archiving and Communication System, IDS 7, Sectra, Linköping, Sweden. In addition, the technical success of the procedure, the duration of the intervention, defined as the period of the groin puncture and the last recorded angiographic series, and peri-procedural complications (such as vessel perforations or off-target embolizations) were assessed.

### Statistical Analysis

Standard descriptive statistics were used to describe all study outcomes. Continuous variables were described with medians and IQRs and categorical variables as absolute and relative frequencies. For group comparisons chi-square and t‑tests were used. Univariable analyses were performed by linear regression analyses with DAP as the dependent variable. The independent variables were the following: age, gender, access route, treated side, treating interventionalist, embolic agent, anesthesia and guiding catheter. Variables with a *p* value < 0.10 in univariable linear regression analyses were included in the multivariable model to avoid excluding potentially relevant predictors due to limited statistical power [12]. In addition, variables that we considered as clinically relevant were included in the final model. Results of the multivariable linear regression model were presented as beta-coefficient together with their 95% confidence intervals and the corresponding *p* values. A *p* value < 0.05 was defined as statistically significant. Statistical analyses were performed with StataMP, Version 18 (StataCorp).

## Results

A total of 112 patients with chronic subdural hematoma who underwent MMA embolization were included in the study. The median (IQR) age was 81 (73–86) and 22 female (20%) and 90 male (80%) patients were included. In 69 patients (62%) particles and in 43 patients (38%) liquid embolic agents were chosen. A total of 32 bilateral embolizations (29%) were performed. In 34 cases (30%), only the right MMA was embolized, and in 46 cases (41%), only the left MMA was embolized. As the guiding catheter, an Envoy was used in 76 cases (68%) and a Simmons 2 in 36 cases (32%). Transradial acces was chosen in 17 cases (15%) and transfemoral access in 95 cases (85%). 70 procedures (63%) were performed under general anesthesia. All included procedures were technically successful. Four patients experienced complications: 1) detection of a prominent MMA-to-ICA collateral pathway, 2) early reflux into the ophthalmic artery, 3) acute right-hemispheric neurological deficit and 4) peri-procedural perforation of a parietal MMA branch. These procedures were not included in the analysis.

**Fig. 1:**
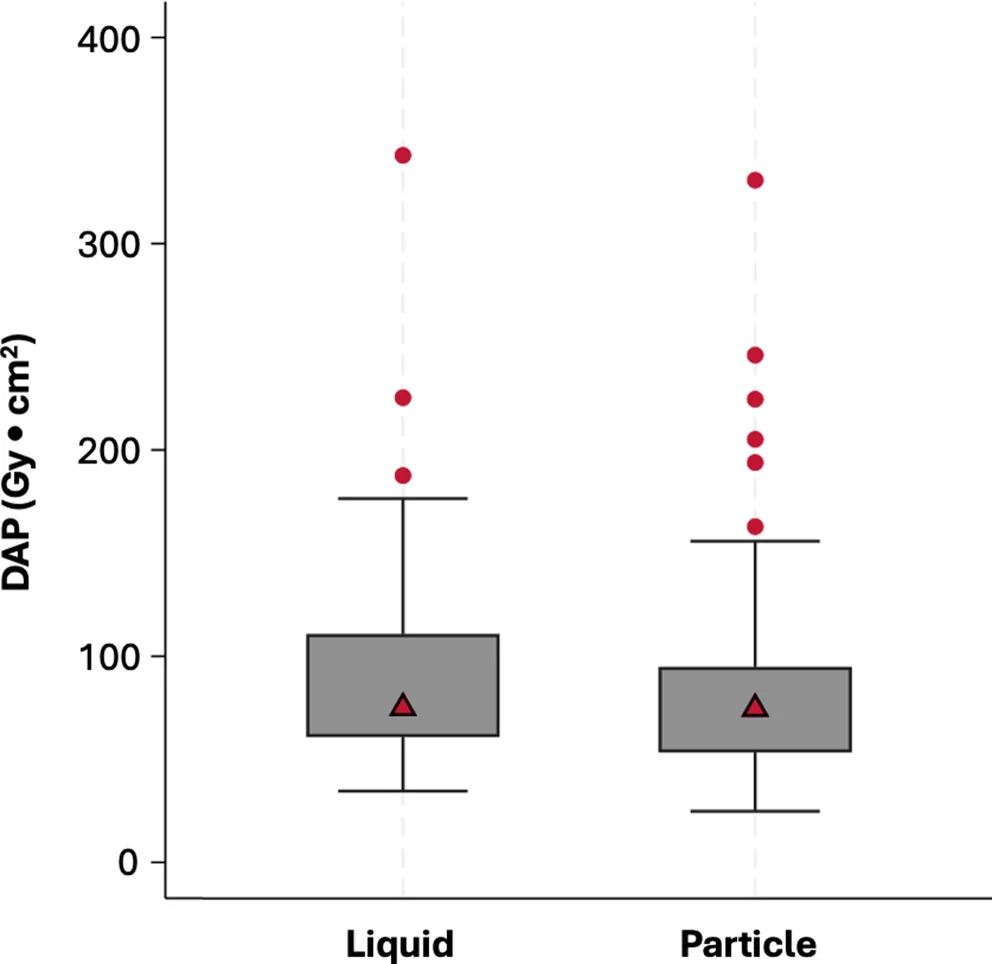
Box plot of radiation exposure by embolic agent. (*DAP* Dose area product)

The overall DAP was 74.7 Gy • cm^2^ (median; IQR, 55.6–102.4), without significant difference between particle and liquid embolic agents (74.6, IQR 53.4–94.9 vs. 75.0, IQR 60.9–110.8; *p* = 0.593). The DAP for unilateral embolization was 65.5 Gy • cm^2^ (median; IQR, 50.6–82.0) and for bilateral embolization 107.6 Gy • cm^2^ (median, IQR 84.1–161.6). Additionally, no significant difference in DAP between particle and liquid embolic agents was observed for either unilateral (60.2, IQR 49.0–80.5 vs. 69.7, IQR 55.7–83.0; *p* = 0.914) and bilateral (104.6, IQR 86.5–150.8 vs. 117.4, IQR 77.8–187.6; *p* = 0.24) procedures (Fig. 1). Table 1 provides detailed overview of patients baseline characteristics.

**Table 1:** Patient demographics and procedural characteristics compared by embolic agent

Radiation Exposure	All Procedures (*n* = 112)	Particle (*n* = 69)	Liquid (*n* = 43)	*P* value
*DAP (Gy* *•* *cm*^*2*^*)*	74.7 (55.6–102.4)	74.6 (53.4–94.9)	75 (60.9–110.8)	0.593
*FT (min)*	21.5 (16–30.5)	22 (16–30)	20 (16–31)	0.918
*Numbers of series (n)*	13 (10–19)	15 (11–20)	12 (9–14)	**0.023**
**Treatment factors**				
*Gender *(female)	22 (20%)	11 (16%)	11 (26%)	0.215
*Age *(years), mean ± SD	78.7 (10)	78.9 (10.6)	78.4 (9.3)	0.26
*Anesthesia* (IA)	70 (63%)	34 (49%)	36 (84%)	< 0.001
*Side* (both)	32 (29%)	21 (31%)	11 (26%)	0.280
Right	34 (30%)	23 (33%)	11 (26%)	
Left	46 (41%)	25 (36%)	21 (48%)	
*Interventionalist *(Most experienced)	16 (14%)	9 (13%)	7 (16%)	0.638
*Guiding Catheter *(Envoy)	76 (68%)	43 (62%)	33 (77%)	0.114
*Arterial Access* (TRA)	17 (15%)	12 (17%)	5 (12%)	0.413

*DAP* Dose area product, *FT* Fluoroscopy time, *TRA* Transradial access, *IA* Intubation anesthesia

### Uni- and Multivariable Analyses

Univariable linear regression analyses revealed no significantly increased radiation exposure for age (β = −0.71 [−1.73–0.32]; *p* = 0.177), sex (β = −15.4 [−41.34–10.53]; *p* = 0.242), interventionalist (β = 1.7 [−27.93–31.32]; *p* = 0.91), anesthesia (β = 11.27 [−10.03–32.58]; *p* = 0.297), guiding catheter (β = 12.07 [−10.01–34.15]; *p* = 0.281), arterial access site (β = 9.41 [−19.43–38.24]; *p* = 0.519) and embolic agent (β = −5.76 [−27.05–15.53]; *p* = 0.593).

A significantly lower radiation exposure was observed only in cases of unilateral embolization compared to bilateral procedures (right: β = −59.2 [−83.2–−35.2]; *p* < 0.001 and left: β = −54.66 [−77.1–−32.23]; *p* < 0.001).

**Fig. 2:**
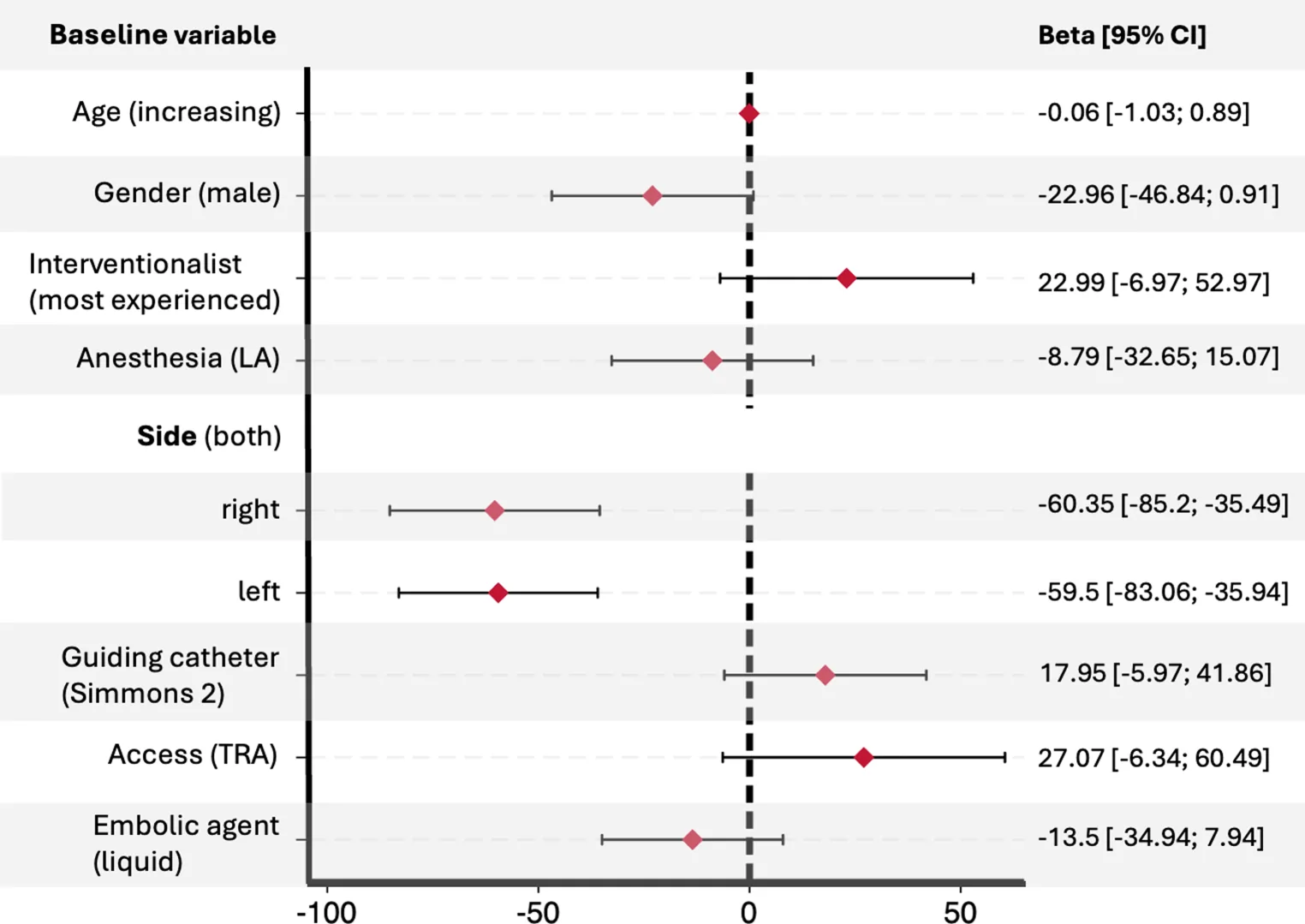
Forest plot of linear multivariable regression analysis for radiation exposure. (*LA* Local anesthesia, *TRA* Transradial access)

In the multivariable linear regression model unilateral embolization was associated with significant lower radiation exposure (right: β = −60.35 [−85.2–−35.49]; *p* < 0.001 and left: β = −59.5 [−83.06–−35.94]; *p* < 0.001). All other covariates were not associated with higher radiation exposure (Fig. 2; Supplement Table 1).

## Discussion

This study investigating the impact of liquid embolic agents and arterial access on radiation exposure during MMA embolization revealed the following main findings: 1) no significant difference in the dose area product was observed between particles and liquid embolic agents; 2) radiation exposure did not differ significantly between transradial and transfemoral arterial access and 3) radiation exposure was driven primarily by the procedure duration, with bilateral embolization yielding significantly higher doses than unilateral embolization.

Currently, there are no diagnostic reference levels specifically established for MMA embolization. When contextualized within the broader neurointerventional landscape, however, our median doses, 65.5 Gy • cm^2^ for unilateral and 107.6 Gy • cm^2^ for bilateral procedures, fall below the diagnostic reference levels of stroke thrombectomy (140 Gy • cm^2^) and endovascular aneurysm treatment (200 Gy • cm^2^). Moreover, published diagnostic reference levels for brain arteriovenous malformation embolization are substantially higher (180–412 Gy • cm^2^), further supporting that MMA embolization generally lies within the lower range of complex neurointerventional procedures. Within this context, our data suggest that MMA embolization can be performed with radiation doses well within the range of other complex neurointerventional procedures, even in an elderly and often multimorbid cSDH population, and may contribute to defining procedure-specific reference levels in future multicenter dose registries.

With regard to arterial access, we did not observe a significant difference in DAP between TRA and TFA. This finding complements recent work comparing radial and femoral access in MMA embolization. A single-center series focusing on elderly patients suggested that TRA is a feasible and safe alternative to TFA and may be associated with advantages such as reduced postoperative delirium without compromising technical success [13]. A large multicenter propensity score–matched study by Salem et al. likewise reported comparable safety and efficacy profiles for TRA and TFA in MMA embolization for cSDH [14]. In addition, Hung et al. demonstrated that TRA is a safe and efficient alternative to TFA in patients undergoing unilateral MMA embolization [15]. Taken together with our results, these data indicate that, under contemporary dose-optimized angiographic protocols, potential access-related differences in catheter stability, navigation, and workflow do not translate into a clinically relevant difference in radiation exposure. Thus, the choice between TRA and TFA in MMA embolization should primarily be guided by patient-specific factors (e.g., vascular anatomy, concomitant cardiac conditions, risk of delirium) and operator experience rather than dose considerations alone.

Importantly, our analysis demonstrates that radiation exposure during MMA embolization is driven mainly by procedural extent. Bilateral embolization was associated with significantly higher DAP compared with unilateral procedures, which is consistent with the longer procedure duration and the need for selective catheterization and embolization of both MMAs. This pattern underscores that cumulative dose in MMA embolization is largely determined by the number of vascular territories treated and the overall fluoroscopy and angiographic run time, rather than by the choice of access route or embolic material. From a practical standpoint, this highlights the importance of meticulous collimation, low-dose fluoroscopy settings, and avoidance of unnecessary angiographic runs, particularly in bilateral cases and in patients who may require repeat embolization [16].

In contrast, we did not detect a significant difference in DAP between particle and liquid embolic agents, either in unilateral or bilateral procedures. This suggests that once stable catheterization of the MMA has been achieved, the embolization phase itself contributes relatively little additional dose compared with the cumulative imaging required for initial vessel selection, anatomical assessment, and post-embolization control angiography. Our finding that DAP did not differ significantly between particle and liquid agents is consistent with previous MMA embolization series [17]. Krothapalli et al. directly compared liquid versus particle materials in patients with chronic subdural hematoma and found no significant differences in radiation dose or fluoroscopy time between groups [18]. Scoville et al. likewise reported similar radiographic and clinical outcomes for particle and liquid embolic agents in nonacute subdural hematomas, without identifying a clear dose advantage for either material [19]. These data, together with our results, suggest that embolic material selection per se is not a major determinant of radiation exposure during MMA embolization and should primarily be guided by anatomical and technical considerations, embolization strategy, and operator familiarity rather than dose concerns.

Moreover, we did not observe a robust association between baseline patient characteristics such as age or sex and DAP in the adjusted model. This suggests that, within a standardized MMA embolization workflow, procedural parameters and case complexity are more relevant targets for dose optimization than demographic factors, which is consistent with previous work emphasizing technical and protocol-based strategies (e.g. frame rate, filtration, projection angles) as the main levers for radiation reduction in neurointervention [20]. Nevertheless, given the advanced age and frequent comorbidities that characterize the cSDH population [21], continued refinement of low-dose imaging protocols and careful justification of bilateral or repeat procedures remain essential to minimize cumulative lifetime radiation exposure for both patients and operators.

This study has several limitations. First, its retrospective single-center design entails a risk of selection bias and limits generalizability to centers with different patient populations, protocols, and angiography systems. Second, the overall sample size was modest and the proportion of TRA cases relatively small, which may have reduced the power to detect subtle access-related differences in radiation exposure. Third, arterial access, embolic agent, anesthetic regimen, and guiding catheter were not standardized but left to operator preference, introducing potential confounding by indication despite multivariable adjustment. Fourth, we did not systematically record certain dose-modifying factors such as detailed body habitus, vessel tortuosity, or exact fluoroscopy and acquisition settings. Also, we did not use personal dose meter to record the operators’ radiation exposure. However, patient’s DAP strongly correlates with the operator’s radiation dose and can therefore serve as a reliable surrogate marker for it [22]. Prospective multicenter studies with standardized imaging protocols and larger TRA cohorts are needed to validate and extend these findings.

## Conclusion

MMA embolization is associated with substantial radiation exposure that is within the range of other complex neurointerventional procedures. Radiation dose was primarily driven by procedural complexity, with higher DAP in bilateral compared to unilateral embolization. The embolization technique and access route were not associated with higher radiation exposure.

## Data Availability

Data are available from the corresponding author upon reasonable request. The data are not publicly available due to patient confidentiality,
